# Elucidating the Multifunctional Roles of Olive Oil By-Products: Phenolic Profiles, Antioxidant Capacity, Probiotic Growth-Promoting and Antimicrobial Activity

**DOI:** 10.3390/foods15193510

**Published:** 2026-10-01

**Authors:** Shaymaa B. Abdulrazzaq, Polina Makarycheva, Stefania Silvi, Marco Zannotti, Rita Giovannetti, Carlos J. García, Rocío García-Villalba, Francisco A. Tomás-Barberán, Dennis Fiorini

**Affiliations:** 1 School of Science and Technology, Chemistry Division, University of Camerino, Via Madonna Delle Carceri 9/B, 62032 Camerino, Italy; shaymaa.abdulrazzaq@unicam.it (S.B.A.); marco.zannotti@unicam.it (M.Z.); rita.giovannetti@unicam.it (R.G.); 2 School of Biosciences and Veterinary Medicine, University of Camerino, Via Gentile III da Varano, 62032 Camerino, Italy; polina.makarycheva@unicam.it (P.M.); stefania.silvi@unicam.it (S.S.); 3 Research Group on Quality, Safety and Bioactivity of Plant Foods, Department of Food Science and Technology, Centro de Edafología y Biología Aplicada del Segura-Consejo Superior de Investigaciones Científicas (CEBAS-CSIC), Espinardo, 30100 Murcia, Spain; cjgarcia@cebas.csic.es (C.J.G.); rgvillalba@cebas.csic.es (R.G.-V.); fatomas@cebas.csic.es (F.A.T.-B.)

**Keywords:** probiotic growth-promoting capacity, phenolic compounds, olive pomace, olive stone, bioactive extract

## Abstract

The olive oil industry generates substantial by-products, notably olive pomace and stones, which present environmental challenges yet harbour untapped bioactive potential. This study investigated the phenolic profiles, antioxidant capacity, and probiotic growth-promoting and antimicrobial activities of olive pomace (OP), olive pomace extract (OPE), olive stones (OS), and olive stone extract (OSE). Phenolic composition, determined by HPLC-DAD/MS, revealed a richer profile and higher phenolic levels in OPE than in OSE, consistent with antioxidant capacity, which was 56.8% higher in OPE (as Trolox equivalents). Probiotic growth promotion was assessed using *Lactiplantibacillus plantarum* IMC 509 and *Lacticaseibacillus rhamnosus* IMC 501^®^, monitoring growth curves, short-chain fatty acid (SCFA) production, total phenolic content (TPC), and antioxidant levels in supplemented culture fluids. Antimicrobial activity was examined against opportunistic pathogens such as *Escherichia coli*, *Pseudomonas aeruginosa*, *Bacillus cereus*, and *Staphylococcus aureus*. All substrates supported probiotic proliferation, and fermenting fluids from *L. plantarum* IMC 509 increased or sustained SCFA production after 28–30 h. OP and OPE significantly enhanced TPC and antioxidant capacity for both strains, whilst OPE and OSE inhibited the tested pathogens. These findings identify olive oil by-products as promising functional food and nutraceutical ingredients, providing preliminary in vitro evidence whilst offering a route to valorise a problematic waste stream.

## 1. Introduction

The olive oil industry is a foundation of the agricultural economies of Mediterranean countries, particularly Spain, Italy, and Greece, where it holds cultural, economic, and health significance [1]. The global demand for olive oil has been on the rise, driven by increasing consumer awareness of its nutritional value, especially its ideal fatty acid composition, high in monounsaturated fatty acids, and the content of bioactives, including phenolic compounds [2]. Phenolic compounds have been extensively studied for their roles in reducing the risk of chronic diseases, such as cardiovascular disease, gut microbiota dysregulation, and inflammatory conditions [3,4]. However, the production of olive oil generates a significant environmental concern due to the high organic load [5]. The olive oil production process leads to large volumes of by-products, primarily olive pomace and vegetation waters [6]. Olive pomace is the olive paste residue left after oil separation, and it contains olive stone fragments and different amounts of water, depending on the production process plant; together, pomace and vegetation water constitute a substantial proportion of the olive biomass [6]. Olive pomace is known to be rich in organic matter and contains a mixture of phenolics, lignins, and fibers [7]. If not properly managed, such by-products can contribute to soil and water pollution, posing a significant environmental risk [6].

The presence of bioactive phenolic compounds in olive pomace and stones presents both a significant opportunity and a challenge for the olive oil industry. The phenolic compounds in waste, including secoiridoid derivatives, are well-established for their potent antioxidant, anti-inflammatory, and antimicrobial properties, making them highly valuable for new applications [8]. Recent advancements in research have shown their potential utilization in the pharmaceutical and food industries, promoting more valorization options of olive by-products rather than downgrading them to waste streams that exacerbate environmental degradation [9]. For example, phenolic compounds extracted from other plant residues, such as pomegranate peels and grape seeds, have been incorporated into food products to enhance their antioxidant properties [10]. This addition helps combat oxidative stress and support cellular health, making them appealing components of health-promoting beverages and functional snacks. Similarly, the phenolic compounds in olive by-products present opportunities for functional foods, nutraceuticals, and pharmaceuticals, emphasizing their ability to provide therapeutic benefits while aligning with the growing consumer demand for natural and sustainable products [11].

Likewise, phenolic compounds are increasingly recognized for their positive ability to influence gut microbiota, functioning as potential prebiotics, stimulating the growth and activity of beneficial microorganisms in the gastrointestinal tract [12]. Hence, they promote the proliferation of beneficial bacteria, such as *Bifidobacterium* and *Lactobacillus* species, while simultaneously exerting inhibitory effects on pathogenic bacteria, including *Escherichia coli* and *Clostridium* species [13,14]. A well-balanced gut microbiota contributes to various physiological functions, such as the enhancement of immune responses, optimization of nutrient metabolism and absorption, regulation of inflammation, and even modulation of neurochemical pathways influencing mental health [14]. For example, the increased abundance of beneficial bacteria facilitated by phenolic compounds can lead to elevated production of short-chain fatty acids (SCFAs), which play a crucial role in maintaining intestinal barrier integrity and mitigating inflammation [15]. Furthermore, phenolic-rich diets have been associated with reduced risks of chronic diseases, including obesity, type 2 diabetes, and cardiovascular disorders, partly through their gut microbiota-mediated effects [16,17]. In this context, a previous study on monovarietal EVOOs from the Marche region reported that these oils selectively stimulated *Lactobacillus* spp. and, most notably in the Raggia cultivar, promoted *Bifidobacterium* populations [4]. Such insights reveal the role of EVOO as a functional food with significant implications for gut health. Another study explored the prebiotic potential of olive pomace as a liquid-enriched powder—mostly a source of phenolics—and pulp-enriched powder, the main source of insoluble dietary fiber, highlighting its ability to enhance the production of SCFAs, including acetate, butyrate, and propionate, during in vitro fermentation. The results demonstrated that olive pomace maintained simulated gut microbiota diversity and favourably modulated the *Prevotella* spp./*Bacteroides* spp. [18]. An eight-week human intervention with olive pomace-enriched biscuits elicited a trend towards increased faecal bifidobacteria and elevated phenolic-acid catabolites in mildly hypercholesterolaemic subjects [19]. Such effects were attributed jointly to the residual insoluble fiber and matrix-bound phenolics of the pomace, supporting its consideration as a functional, prebiotic-type ingredient [20]. By contrast, olive-stone-derived material remains far less investigated for prebiotic effects or probiotic growth-promoting assessment, a gap that directly motivates the present study comparing also olive stone and its phenolic extract. In addition, olive-derived phenolics, particularly hydroxytyrosol and oleuropein, exhibit pronounced antibacterial activity against a broad spectrum of foodborne pathogens, with the oils from olive fruits showing strong bactericidal action, this effect being generally greater against Gram-positive than against Gram-negative bacteria [21]. This activity has been mechanistically linked to the binding of hydroxytyrosol and oleuropein within the polyphenol pocket of a bacterial enzyme responsible for energy adenosine triphosphate (ATP) production, thereby disrupting the cell’s energy supply [22].

Building on this gap, the present study provides an integrated assessment of two EVOO-production by-products (olive pomace (OP) and olive stones (OS)) together with their hydroalcoholic extracts (OPE, OSE), combining probiotic-promoting activity, antimicrobial, and chemical characterization within a single experimental framework. The probiotic growth-promoting activity of each treatment was evaluated on two characterized commercial strains, *Lacticaseibacillus rhamnosus* IMC 501^®^ and *Lactiplantibacillus plantarum* IMC 509, by monitoring growth curves alongside SCFA production, total phenolic content (TPC), and antioxidant levels in the resulting culture fluids; these strains were selected for their established probiotic functionality and prior use in our laboratory, ensuring reproducibility of the experimental approach [23,24,25]. In parallel, the antimicrobial activity of OPE and OSE was tested against opportunistic Gram-negative and Gram-positive pathogens of food safety and clinical relevance, *E. coli* ATCC 13706, *Pseudomonas aeruginosa* DSM 1117, *Bacillus cereus* ATCC 9634, and *Staphylococcus aureus* ATCC 25923. The phenolic profiles of the extracts were compared by HPLC-DAD/MS together with their TPC and DPPH radical-scavenging activity, and the elemental composition of OP and OS was determined by ICP-MS, given the known influence of minerals on microbial growth [7,26].

## 2. Materials and Methods

### 2.1. Materials

Folin–Ciocalteu reagent, 2-diphenyl-1-picrylhydrazil (DPPH), Trolox, and *i*-caproic acid were purchased from Sigma-Aldrich (Milan, Italy). HPLC-grade methanol and *n*-hexane were purchased from Sigma-Aldrich (Milan, Italy). Water (resistivity above 18 MΩ·cm) was obtained from a Milli-Q SP Reagent Water System (Millipore, Bedford, MA, USA). All solvents and solutions were filtered through a 0.45 μm PTFE filter from Supelco (Bellefonte, PA, USA) before use in the HPLC-DAD-MS analysis. Sulfuric acid and ethanol (96% *v*/*v*) were purchased from Carlo Erba (Milan, Italy)), and ethyl ether from J.T. Baker (Phillipsburg, NJ, USA). The analytical standards of hydroxytyrosol, tyrosol, oleuropein, luteolin, and apigenin were purchased from Extrasynthese (Genay, France). *p*-Coumaric acid and pinoresinol were purchased from Sigma-Aldrich (Milan, Italy).

### 2.2. Sample Preparation and Extraction Process

#### 2.2.1. Origin and Preparation of the Olive Oil By-Products

The by-products, namely OP and OS, originated from a single monovarietal (Raggia) production batch supplied by one mill (‘Il Frantoio del Piceno’, Montegranaro, FM, Marche region, Italy). The olives were harvested on the 11th of November 2023 and soon processed; immediately after oil separation, the by-products were collected and transported to the laboratory under refrigeration. Raggia was selected since it is one of the dominant autochthonous cultivars of the Marche region and, in a previous study [4], showed a high bifidogenic activity among local monovarietal EVOOs, making its by-products a rational focal matrix. The oil was obtained by means of a two-phase olive mill plant. After cleaning and washing the olives with cold water, they were crushed by a knife mill, and the olive paste obtained was transferred by pumps into vertical malaxation cylinders where malaxation was performed at cold (20–22 °C), under vacuum, for 20 min. The malaxed paste was then transferred by pumps into decanters, providing the separation of the oil from the olive pomace. Ultimately, a machine separated the stone parts (having dimensions of 2–3 mm) from the pomace. These two separated products, OP and OS, were used in this study. Olive stones were layered to allow complete drying before being stored at room temperature in closed polyethylene bags. Pomace was frozen at −20 °C, and then lyophilized utilizing an Edwards DO1 freeze-dryer (chamber volume: 23 L), for 72 h at a condenser temperature of −50 °C and a chamber pressure of 0.16–0.2 mbar, subsequently sieved to yield a fine pomace powder (<850 µm), and stored at −20 °C until further analytical procedures. Similarly, OS was powdered using a laboratory mill (IKA^®^ Tube Mill control, IKA, Staufen, Germany) applied in repeated 1 min cycles, with pauses between cycles to prevent sample heating, until a fine, homogeneous powder was obtained. The powder was then sieved to <850 µm to optimise subsequent extraction efficiency. For each substrate (OP and OS), approximately 1 kg was sieved and well homogenized in order to ensure good representativeness of each aliquot used for the subsequent extractions and analyses.

#### 2.2.2. Hydroalcoholic Extraction

Hydroalcoholic extracts were obtained by homogenizing 20 g of each sample in 100 mL of an 80:20 (*v*/*v*) ethanol/water solvent, employing an Ultra-Turrax homogenizer (DI 25 basic, IKA, Staufen, Germany), with the rotational speed increased stepwise from 8000 to 24,000 rpm (peripheral speed ≈ 7.5–22.6 m/s) over 10 min, followed by continuous magnetic stirring (Fisher Scientific FB15002, Waltham, MA, USA) at room temperature for 6 h. The resultant suspensions were filtered with filter paper (Whatman No. 1, Cytiva, Buckinghamshire, UK; pore size: 11 µm), and the solvent was removed under reduced pressure via rotary evaporation. The residual extracts were reconstituted in 10 mL of a 20:80 (*v*/*v*) ethanol/water solvent and maintained at −20 °C until required for analyses.

The 80:20 (*v*/*v*) ethanol/water composition was selected because hydroethanolic solvents in the 70–80% ethanol range are widely reported as optimal for recovering the principal olive phenolics, offering a favourable balance between extraction efficiency and food-grade, generally recognised as safe (GRAS) solvent status [27,28]. The lower ethanol proportion (20:80, *v*/*v*) used for reconstitution was chosen to keep the phenolics in solution while minimising ethanol carry-over into the microbiological assays, where residual solvent could otherwise confound the measured probiotic and antimicrobial responses.

Before any analytical evaluation, 0.5 mL aliquots of each extract (OPE and OSE, obtained from OP and OS respectively) underwent a defatting procedure (intended solely for spectrophotometric and analytical assays). This involved the addition of 0.5 mL of hexane, followed by vortex mixing for 1 min and centrifugation (ALC 4218 benchtop centrifuge, ALC, Milan, Italy) at 1000× *g* for 10 min. at room temperature. The supernatant hexane phase, containing lipophilic constituents, was aspirated and discarded. This defatting protocol was repeated twice to ensure the comprehensive removal of non-polar fractions.

### 2.3. Characterization of Olive Oil By-Products

#### 2.3.1. Phenolic Profiles of Extracts by HPLC-DAD/MS Analysis

Two independent replicates of each extract (OPE and OSE), obtained by independent extractions, were analysed. The reconstituted extracts were diluted four-fold with ethanol: water (20:80, *v*/*v*) solvent. The method for analyzing the phenolic profiles of the samples was adapted from previously published methods [29,30]. Briefly, high-performance liquid chromatography coupled with diode–array detection and electrospray ionization mass spectrometry (HPLC-DAD/MS) was employed for the analysis, an Agilent 1260 Infinity II (Santa Clara, CA, USA) instrument, made from an autosampler, a quaternary solvent pump, with a diode–array detector (DAD) and a single quadrupole mass spectrometer detector (MSD) equipped with an electrospray ionization (ESI) source operating in negative ionization (NI) mode. The separation was achieved on a Synergy Polar reverse phase (RP) (250 mm × 4.6 mm, 4 μm) analytical column from Phenomenex (Cheshire, UK). The mobile phase for HPLC-DAD/MS analyses was water with 0.1% formic acid (A) and methanol/isopropanol 90:10 *v*/*v* with 0.1% formic acid (B) working in the gradient mode at a flow rate of 1 mL min^−1^. The solvent composition varied as follows: 0 min, 30% B; 0–40 min, 60% B; 40–50 min 95% B; then, the column was reconditioned. The column temperature was set at 35 °C, and the injection volume was 10 μL. HPLC-DAD analysis used for the quantification was performed by monitoring different wavelengths: 280 nm for hydroxytyrosol, tyrosol, verbascoside, secoiridoids derivatives, pinoresinol, and acetoxypinoresinol; 310 nm for *p*-coumaric acid, 325 nm for ferulic acid, 338 nm for apigenin, and 350 nm for luteolin. Tyrosol, hydroxytyrosol, oleuropein, pinoresinol, luteolin, and apigenin were quantified by building calibration curves with the corresponding standards. The other phenolics were tentatively identified by MS according to their molecular ion and to the literature, and quantified in terms of phenolics composing their structures, i.e., tyrosol and hydroxytyrosol complexes were quantified in terms of tyrosol and hydroxytyrosol, respectively. Comselogoside was quantified in terms of *p*-coumaric acid; acetoxypinoresinol was quantified in terms of pinoresinol. In MS, for the purposes of identification, the ion source was operating in NI mode. The scan mode ran from 50 to 1500 *m*/*z*. The drying gas flow was 13 L/min. Nebulizer pressure was 60 psi, drying gas temperature was 350 °C, and capillary voltage was 3000 V. Mass scan range was set in the range of *m*/*z* 50–1500. Extract ion chromatogram (EIC) from total ion chromatogram (TIC) was used for the analysis.

#### 2.3.2. Quantitative Elemental Composition Analysis by ICP-MS

The powdered freeze-dried OP and OS samples were previously mineralized via acid digestion using a Berghof Speedwave 4 microwave digestion system in triplicate (Berghof, Eningen, Germany). The digestion was carried out in Teflon vessels with 4 mL of 30–32% H_2_O_2_, 1 mL of 65–69% HNO_3_, 0.02 g of sample powder, and 50 µL of a solution containing 2 µg/L of Au, Be, and Ru, used as recovery standards. This solution was prepared from single-element standard solutions (1 g/L, ICP-MS grade, Fluka Analytical, Merck, Darmstadt, Germany).

After digestion, the mineralized samples were transferred to plastic tubes and diluted with the addition of 5 mL of ultrapure water (resistivity 18.2 MΩ cm), produced using a Millipore Milli-Q system (Millipore, Molsheim, France). The prepared samples were then analysed using an Agilent 7500cx ICP-MS system (Agilent Technologies, Santa Clara, CA, USA).

ICP-MS operating conditions were as follows: radiofrequency (RF) power 1550 W, carrier gas flow rate 0.9 L/min, sample depth 7 mm, nebulizer pump speed 0.1 r.p.s. and spray chamber temperature 2 °C. The analysis was performed in NoGas/He mode to effectively reduce polyatomic interferences by using a collision cell.

A solution containing ^45^Sc, ^115^In, ^140^Ce, and ^209^Bi (10 µg/L) was used as the internal standard, prepared from single-element standard solutions (1 g/L, ICP-MS grade, Fluka Analytical, Merck, Darmstadt, Germany).

Calibration for trace elements was performed using standard solutions at the following concentrations: 0.01, 0.10, 1.00, 5.00, 10.0, 50.0, 100.0, and 500.0 ppb. For macro-elements (Na, Mg, P, S, K, Ca), calibration standards were prepared at: 0.1, 0.50, 1.00, 2.50, 5.00, 10.0, 25.0, and 50.0 ppm. All calibration standards were prepared using 10 mg/L ICP-MS calibration solutions supplied by Agilent Technologies (Santa Clara, CA, USA).

### 2.4. Assessment of Antimicrobial Activity

The assessment of antimicrobial activity was performed to: (i) exclude that the extract by-products had inhibitory activity on the probiotic bacterial strains; (ii) evaluate their inhibitory capacity on the pathogens tested.

#### 2.4.1. Bacterial Strains and Growth Conditions

*Lactiplantibacillus plantarum* IMC 509 and *Lacticaseibacillus rhamnosus* IMC 501^®^ were supplied by Synbiotec srl (Camerino, Italy). Both strains were activated on de Man, Rogosa, and Sharpe (VWR, Leuven, Belgium) broth or agar plates (MRS). *L. plantarum* IMC 509 was grown under anaerobic conditions (AnaeroGen™, Thermo Scientific, Tokyo, Japan), and *L. rhamnosus* IMC 501^®^ under aerobic conditions at 37 °C for 48 h.

Four pathogenic strains *E. coli* ATCC13706, *Ps. aeruginosa* DSM1117, *B. cereus* ATCC9634, *S. aureus* ATCC25923, were grown in Tryptic Soy Broth (TSB, VWR, Belgium) and Tryptic Soy Agar (TSA, VWR, Belgium) for 24 h under aerobic conditions.

#### 2.4.2. Minimal Inhibitory Concentration

Isolated colonies of each probiotic and each pathogen strain were used to standardize the bacterial suspension using a spectrophotometer (UV-1601, Shimadzu, Kyoto, Japan) at OD_600 nm_ = 0.1 or 0.04 absorbance for pathogens and probiotics, respectively. A minimal inhibitory concentration (MIC) experiment was organized using 96-well ELISA microplates. Only the extracts were used in this test due to the difficulty of dissolving the olive pomace and stone. Descending concentrations of OPE and OSE were loaded into each well, starting from the initial concentration of 137 mg/mL for OPE and 32 mg/mL for OSE. The solvent 20:80 (*v*/*v*) ethanol/water (named EWA) was used as a control. For each strain, the suitable culture medium was added to each well to obtain a total volume of 100 µL. Lastly, 50 µL of bacterial strain suspension was added to each well, excluding the negative control. Three independent series of wells were prepared for each experiment. ELISA plates were incubated for 24 h in aerobic/anaerobic conditions. At the end of the incubation, results were read using the µQuant™ Universal Microplate Spectrophotometer (BIO-TEK^®^, Shoreline, WA, USA) at OD_600 nm_.

Percentage of inhibition was calculated using the following formula:

$$\%\;\mathrm{inhibition}\;=\;100\;\times \;(1\;-\;\frac{\mathrm{Sample}\;-\;\mathrm{Min}1}{\mathrm{Control}\;-\;\mathrm{Min}2})$$

where:

*Min1* is the blank with the substance at the same concentration as the value.

*Min2* is the blank without substance.

The MIC test evidenced that the 1% concentration of EWA did not affect the growth pattern of the probiotic strains. Further on, 1% of the extract was used in the batch culture experiment.

### 2.5. Batch Culture of Probiotic Strains with Olive Oil By-Product Supplementation

Batch cultures of probiotic strains were set up using 30 mL of MRS broth supplemented with 1% concentration of OP and OS (as lyophilized powders, previously checked for absence of microbial contaminants). OPE and OSE were used dissolved in solvent 20:80 (*v*/*v*) ethanol/water.

Before the start of the experiment, probiotic bacterial cell inoculum was standardized using a spectrophotometer (Shimadzu, Japan) (OD_600 nm_ = 0.045 absorbance). All cultures were left to incubate at 37 °C with shaking (170 rpm) in the water bath (PBI International, Brussels, Belgium).

The MRS was chosen as a constant nutritional base, whilst the olive by-products represented the experimental variable. Thus, any differences in probiotic growth, SCFA production, total phenolic content, and antioxidant capacity can therefore be attributed to the effect of the tested by-products and not to variations in the composition of the culture medium.

Bacterial growth was monitored by measuring pH at two-hour intervals from T0 to T12 and from T24 until the pH value was stable, where the notation Tn denotes the time, in hours, elapsed from inoculation (e.g., T0 = moment of culture inoculation and T30 = culture reaches plateau phase). Viable bacterial counts were performed at T0, T9, T24, and T28 or T30 (based on the pH value measurements) to monitor bacterial growth and plot growth curves. The bacterial enumeration at each time point was performed in triplicate. As a control, the *L. plantarum* IMC 509 and *L. rhamnosus* IMC 501^®^ were inoculated in MRS broth and in MRS supplemented with the solvent (EWA). For each bacterial culture, the bacterial cell increment (Δ) and the growth rate were considered by applying the following Formulas (1) and (2):

(1) $$\mathrm{Bacterial}\;\mathrm{growth}\;\mathrm{increment}\;(\Delta )\;=\;\log\;{\mathrm{CFU}}_{T_{\mathrm{final}}}^{-\mathrm{mL}}\;-\;\log{\mathrm{CFU}}_{T_{0}}^{-\mathrm{mL}}$$

(2) *Growth rate* (µ) = Δ/*Hours_Tfinal_*

In addition, for each compound in relation to every single probiotic strain, the prebiotic activity score was calculated by applying the formula from [31] with modifications (3):

(3) $$\mathrm{Prebiotic}\;\mathrm{activity}\;\mathrm{score}\;(\mathrm{PAS})\;=\;\frac{\Delta_{\left(\mathrm{tested}\;\mathrm{compound}\;\mathrm{prebiotic}\right)}}{\Delta_{\left(\mathrm{control}\;\mathrm{IMC}\right)}}$$

where:

Δ_(_*_tested compound_*_)_ is the probiotic strain growth-increment in the medium supplemented with the tested compound.

Δ_(_*_control IMC_*_)_ is the probiotic strain growth-increment in the medium without any tested compound.

### 2.6. SCFA Extraction and Analysis in Fermentation Fluids

The analysis of SCFAs in bacterial culture fluid samples at the final time point (T28 or T30 depending on the strain) was conducted following a method reported in the literature [32], slightly modified. For each experiment, two replicates (two independent fermentation fluids) were analysed. An aliquot of 250 mg of culture fluid was acidified with 50 µL of 50% (*w*/*v*) sulfuric acid and vigorously mixed using a vortex for 1 min. The acidified solution was subsequently extracted with 800 µL of diethyl ether and centrifuged at 2800× *g* for 5 min. The supernatant organic phase was carefully transferred to a separate 4 mL vial. This extraction procedure was repeated three times, yielding a total of 2400 µL of organic phase. To eliminate residual moisture, a small quantity of anhydrous sodium sulfate (several milligrams) was added to the pooled organic phase. Finally, 0.5 µL of the resulting organic phase was directly injected into a gas chromatograph (GC) for analysis, without prior derivatization of the SCFAs.

The analysis was conducted using an Agilent Technologies 6850 GC equipped with a split/splitless injector and a flame ionization detector (FID) (Santa Clara, CA, USA) as described previously [32]. Briefly, the chromatographic separation was performed on a nitro-terephthalic acid-modified polyethylene glycol (PEG) capillary column (DB-FFAP, 25 m length, 0.25 mm internal diameter, and 0.25 µm film thickness, Agilent Technologies, Santa Clara, CA, USA). The GC injector was set at a temperature of 280 °C and operated in splitless mode, with a splitless time of 3 min. The oven temperature program began at 40 °C (held for 3 min), increased at a rate of 20 °C/min to 160 °C, followed by an increase at 40 °C/min to 245 °C, where it was held for 1.87 min. The total running time was 13 min. Hydrogen was used as carrier gas, with a flow rate of 3.70 mL/min. The FID was maintained at a temperature of 250 °C.

### 2.7. Determination of the Total Reducing Capacity of Extracts and Fermentation Fluids

Folin–Ciocâlteu assay, adapted from the protocol described in the literature by [33], was used to determine the total reducing capacity of the extracts, expressed as Total Phenol Content (TPC) assuming that the most abundant substances responsible for the reducing capacity are phenolics. OPE was diluted at 1:30 (*v*/*v*) and OSE at 1:20 (*v*/*v*), each with ultra-pure water. For the fermentation culture fluids, samples collected at the initial and final sampling points were diluted 1:10 (*v*/*v*) with ultra-pure water. Two independent replicates of each extract (OPE and OSE), obtained by independent extractions, each from an independent fermentation fluid, were analysed.

Subsequently, 1.5 mL of each diluted sample was mixed with 0.5 mL of Folin–Ciocalteu reagent and 0.5 mL of a 7.5% (*w*/*v*) sodium carbonate (Na_2_CO_3_) solution in a 10 mL volumetric flask. The mixture was then adjusted to the final volume with DW, incubated in the dark for four hours at room temperature to allow the reaction to proceed to completion, ensuring standardized conditions for spectrophotometric analysis. For the calibration curve used for determining TPC, a stock solution of gallic acid at a concentration of 2000 mg/L was prepared in methanol. A series of six standard solutions was subsequently prepared by diluting the stock solution to achieve gallic acid concentrations ranging from 50 ppm to 700 ppm. For each calibration point, 0.2 mL of the diluted standard solution was combined with 1.2 mL of methanol, 0.5 mL of Folin–Ciocâlteu reagent, and 0.5 mL of 7.5% Na_2_CO_3_. The mixture was then diluted to a final volume of 10 mL using DW. The prepared solutions were incubated in the dark for four hours at room temperature to allow the reaction to proceed to completion. The absorbance of each sample was measured at 765 nm using a Cary 8454 UV-Vis spectrophotometer (Agilent Technologies, Woburn, MA, USA), with the corresponding solvent used as the blank. The TPC of each sample was calculated by comparing its absorbance to the standard calibration curve constructed from the gallic acid standard. Results were expressed as gallic acid equivalents (GAE) in ppm, reflecting the phenolic content of the samples based on the calibration curve.

### 2.8. Antioxidant Scavenging Capacity of Extracts and Fermentation Fluids

The antioxidant activity of the samples was evaluated using the 2,2-diphenyl-1-picrylhydrazyl (DPPH) free radical scavenging assay, adapted from established protocols [34,35]. Two independent replicates of each extract (OPE and OSE), obtained by independent extractions, were analysed. Briefly, a DPPH stock solution was prepared by dissolving 3.94 mg of DPPH powder in 100 mL of methanol, yielding a stable purple solution. For the blank, 0.5 mL of DW was mixed with 4.5 mL of methanol to establish a baseline. The control consisted of 0.5 mL of DW and 4.5 mL of the DPPH solution, providing a reference for maximum radical absorbance. For sample analysis of the extracts, each was subjected to defatting as mentioned previously in Section 2.2. The OPE and OSE were diluted at 1:20 *v*/*v*. Similarly, for the determination of the antioxidant capacity in bacterial culture fluids, 0.5 mL of samples collected at the initial and final time points were diluted at a 1:10 (*v*/*v*) ratio with DW. Sample analysis was conducted by mixing 0.5 mL of the diluted sample with 4.5 mL of the DPPH solution. All mixtures were incubated in the dark at room temperature for 30 min to allow for radical scavenging, after which the absorbance was measured at 517 nm using a UV-Vis spectrophotometer. The decrease in absorbance relative to the control was used to quantify the antioxidant capacity of the samples, reflecting their ability to neutralize DPPH radicals. Results were presented as a percentage of DPPH inhibition and Trolox ppm. The percentage inhibition of DPPH radical scavenging activity was calculated using the following formula:

$$\%\;\mathrm{Inhibition}\;\mathrm{of}\;\mathrm{DPPH}\;=\;(\frac{\mathrm{Abs}\;\mathrm{control}\;-\;\mathrm{Abs}\;\mathrm{sample}}{\mathrm{Abs}\;\mathrm{control}})\;\times \;100\%$$

where *Abs control* represents the absorbance of the control DPPH, and *Abs sample* represents the absorbance of the sample after reaction with DPPH.

### 2.9. Statistical Analysis

All data are expressed as mean ± standard deviation (SD) of *n* = 2 or 3 independent replicates, depending on the type of analysis, as indicated in the previous sections. The four samples compared in this study, namely, olive pomace (OP), olive stone (OS) and their corresponding extracts (OPE and OSE), were obtained from a single EVOO production. For the chemical analyses, the large quantity of starting material (1 kg of each raw sample), together with thorough homogenisation and reduction to a small, uniform particle size, ensured the representativeness of each aliquot and a low variability between replicates, which was confirmed in preliminary tests with several replicates; a limited number of replicates was therefore considered sufficient for these analyses.

For the microbiological study, designed to compare the four substrates across two probiotic strains and several endpoints (growth, SCFA production, total phenolic content and antioxidant capacity) in order to identify the most promising matrices for subsequent studies, the low dispersion between replicates, reflected in the SD values reported throughout, supports the internal consistency of the measurements.

Statistical tests were selected according to the data structure. Probiotic growth was compared between two treatments using a two-tailed *t*-test. The total phenolic content and DPPH antioxidant capacity of the fermenting fluids, compared among the four treatments across sampling times (T0–T28), were analysed by two-way ANOVA (treatment × time), followed by Tukey’s multiple-comparisons test. Short-chain fatty acids (SCFA), compared among treatments at a single time point, were analysed by one-way ANOVA, followed by Tukey’s test. A probability value of *p* < 0.05 was taken as the threshold for statistical significance (* *p* < 0.05, ** *p* < 0.001, *** *p* < 0.0001, **** *p* < 0.00001). All statistical analyses were performed in GraphPad Prism^®^ (version 8.4.3, GraphPad Software, San Diego, CA, USA).

## 3. Results

### 3.1. Characterization of Olive Oil By-Products

#### 3.1.1. Polar Phenolic Compounds Analysis by HPLC-DAD/MS

The phenolic profiles of OPE and OSE were characterized using high-performance liquid chromatography coupled with diode–array detection and mass spectrometry (HPLC-DAD/MS). The main phenolics detected are reported in Table 1. The compounds identified using authentic reference standards were hydroxytyrosol, tyrosol, oleuropein, pinoresinol, luteolin, and apigenin, whereas the others were considered as tentatively identified based on molecular mass data and according to several other studies reporting their presence in the same olive-derived matrices [36,37,38,39,40]. OPE and OSE revealed a diverse range of phenolic compounds, with notable differences especially in abundance. OPE exhibited a richer and more varied phenolic profile, with verbascoside (representing approximately 60% of the total phenolics detected), secoiridoid derivatives, and other hydroxytyrosol and tyrosol complexes as the most abundant compounds. Minor phenolics detected in OPE included lignans (acetoxypinoresinol and pinoresinol) and flavonoids (luteolin and apigenin).

OSE displayed a less diverse phenolic profile with generally lower overall abundances, except for lignans and flavonoids, which were in this case slightly more abundant compared to OPE (Figures S1 and S2 in the Supplementary Material).

#### 3.1.2. Total Phenolic Content (TPC) and Antioxidant Levels of Extracts

The TPC and antioxidant capacity were assessed in hydroalcoholic extracts derived from OP and OS. TPC, expressed as mg of GAE/mL, revealed higher phenolic content in OPE at 1548.3 ± 443.4 mg GAE/mL compared to OSE at 940.8 ± 127.4 mg GAE/mL. Meanwhile, the antioxidant capacity, assessed via the DPPH radical-scavenging assay and expressed as Trolox equivalents in ppm, was also significantly greater in OPE at 4365.2 ± 12 ppm Trolox compared to OSE at 2788.7 ± 8.8 ppm Trolox. These results indicate that OPE possesses a higher concentration of phenolic compounds and greater antioxidant activity than OSE, reflecting potential differences in their chemical composition and, consequently, biological activity.

#### 3.1.3. Elemental Composition Analysis by ICP-MS

The elemental composition of OP and OS was quantified using inductively coupled plasma mass spectrometry (ICP-MS), providing a comprehensive mineral profile for each olive oil by-product. Results are expressed as mean concentrations (mg/kg) ± standard deviation (*n* = 3), as detailed in Table 2. The elemental composition of OP and OS is characterized by six main elements, K, Ca, P, S, Na, and Mg, that represent 99.7% and 99.4% of the total mass of OP and OS, respectively. The elements analyzed by ICP-MS are all detected except for Tl, Hg, Rb, Mo, Sn, Sb, Be, Au, and Ru; considering that, the last three elements are used as recovery standards. Elemental analysis results obtained via ICP-MS are graphically illustrated in Figure 1 and quantitatively detailed in Table 2. Analyzing the OP and OS samples, the element concentrations for the macro and microelements are highly concentrated in OP with respect to the OS. Elemental profiles determined by ICP-MS showed that K was the predominant element in both matrices, but with a notably higher percentage in OP (81.8%) compared to OS (51.6%). The absolute Ca content was much higher in OP; however, the relative concentration was higher in OS (35.8%) than in OP (8.6%). In lower concentrations, elements such as P, S, Na and Mg were detected in both samples, with proportions ranging between 0.5% and 5% of the total elemental composition.

Regarding the microelements, B and Fe are the most abundant; other notable elements include Mn, Zn, Sr and Cu. Minor contributions are observed for Ba and the other microelements [41].

### 3.2. Assessment of Antimicrobial Effect

The antimicrobial effect of the two extracts, evaluated by MIC assay, revealed that the substrates of interest did not inhibit the growth of the two probiotic strains at any of the concentrations tested. In contrast, the extracts in our study had an inhibitory effect on the growth of the selected pathogenic strains. Interestingly, OSE at a concentration of 0.2 mg/mL showed an outstanding capacity to inhibit the growth of *Ps. aeruginosa* DSM1117 (with 67.1 ± 7.3% inhibition). The value of 6.7 mg/mL was the lowest OPE concentration that effectively inhibited two of the opportunistic pathogenic strains *E. coli* ATCC 13706 and *B. cereus* ATCC 9634, with a % inhibition of 76.1 ± 13.4 and 69.4 ± 0.7, respectively (Table 3).

### 3.3. Growth Curves of Probiotic Bacteria in the Presence of Olive Oil By-Products and Extracts

The growth of *L. plantarum* IMC 509 and *L. rhamnosus* IMC 501^®^ was evaluated at the presence of OP and OS (added as lyophilized powder at 1%), and their hydroalcoholic extracts (OPE and OSE, respectively) at time intervals until 28/30 h (T0-T28/30) as shown in Figure 2. The *L. plantarum* IMC 509 and *L. rhamnosus* IMC 501^®^ growth curves in MRS broth (IMC 501^®^ and IMC 509) and their culture in MRS broth supplemented with ethanol: water solvent (EWA_IMC501 and EWA_IMC509) were the controls.

All the compounds used as supplements supported probiotic growth, with varying efficacy depending on the substrate and bacterial strain (Figure 2 and Table 4). Both probiotic strains exhibited the highest growth rates in the presence of OPE, gaining 7.3 and 7.0 in terms of log value, for IMC 501^®^ and IMC 509 respectively, followed by OSE, OP, and OS. Extracts consistently promoted higher bacterial growth compared to their unextracted counterparts. *L. plantarum* IMC 509 showed a higher growth rate with OPE (0.25 µ) and OSE (0.22 µ), while *L. rhamnosus* IMC 501^®^ displayed similar trends for OPE (0.24 µ) and OSE (0.23 µ). Growth curves indicated a logarithmic increment in bacterial counts, peaking at T28/30 across all supplements, with OPE yielding the highest viable counts for both strains (Table 4).

Furthermore, OP and OS showed the highest prebiotic activity score when supplemented with *L. plantarum* IMC 509 (2.1 and 2.0, respectively).

### 3.4. Short-Chain Fatty Acid Production in Fermentation Fluids

The production of SCFAs, from acetic (C2) to hexanoic acid (C6), was assessed in bacterial culture fluids inoculated with *L. plantarum* IMC 509 and *L. rhamnosus* IMC 501^®^ in the presence of all the supplemented substrates over time intervals up to 28/30 h (T28/30) as shown in Figure 3. Acetic and valeric acid (C5) were the only two SCFAs produced in a relevant amount. Acetate was the predominant SCFA across all substrates, with comparable levels observed in OP, OPE, OS, and OSE for both bacterial strains.

For acetate (Figure 3A,B), the four treatments OP, OPE, OS, and OSE sustained production at levels comparable to, and in several cases exceeding, the respective growth controls, confirming that neither the whole by-products nor their extracts impaired the fermentative capacity of the two probiotics. In *L. rhamnosus* IMC 501^®^ (Figure 3A), acetate was essentially uniform across all conditions with no significant differences, indicating compatibility of the treatments with strain growth. In *L. plantarum* IMC 509 (Figure 3B), acetate accumulation was higher overall, and the supplemented cultures reached concentrations significantly above the IMC 509 growth control, with OPE and OSE giving the strongest response; this demonstrates that the treatments not only preserved but modestly enhanced the primary fermentative growth and/or general output of this strain.

Valerate (Figure 3C,D) revealed a clearer substrate-dependent effect, with the whole by-products OP and OS eliciting the highest C_5_ output in both strains. In *L. rhamnosus* IMC 501^®^ (Figure 3C), OS and OP induced significantly greater valerate than the IMC 501^®^ growth control (*p* < 0.05), whereas the corresponding extracts OSE and OPE yielded lower, control-comparable levels. The same trend was reproduced by *L. plantarum* IMC 509 (Figure 3D), where OP and OS again produced the highest valerate and all four supplements exceeded the IMC 509 growth control. Collectively, both probiotic strains remained metabolically active on every olive by-product treatment, with the fiber-rich whole materials (OP, OS) favouring valerate production and all four supplements maintaining or enhancing acetate output relative to the growth controls. These findings confirm that OP, OPE, OS and OSE are compatible with, and in several instances stimulatory to SCFA production of *L. rhamnosus* IMC 501^®^ and *L. plantarum* IMC 509.

### 3.5. Total Reducing Capacity and Antioxidant Levels of Fermentation Fluids

Total reducing capacity, measured with the Folin–Ciocalteu assay and expressed in terms of total phenolic content (TPC) and antioxidant scavenging capacity, in terms of the DPPH radical scavenging assay, of bacterial culture fluids, derived from the incubation of *L. plantarum* IMC 509 and *L. rhamnosus* IMC 501^®^ with the four supplements, exhibited changes from T0 to final (T28/30) time points as shown in Figure 4. The total reducing capacity, measured as GAE in ppm, increased across some treatments, with OP and OPE showing the most substantial rises for *L. plantarum* IMC 509 (*p* < 0.001, *p* < 0.001, respectively). The increase may reflect residual or released phenolics, microbial metabolites, or medium-derived interference; targeted phenolic profiling before and after fermentation would be required to confirm that specific aglycones (e.g., hydroxytyrosol from its glucosides, or verbascoside-derived metabolites) drive the effect. Similarly, *L. rhamnosus* IMC 501^®^ displayed a similar trend in TPC change, with OP providing a significant change (*p* < 0.05).

Antioxidant capacity, assessed via the DPPH radical scavenging assay and expressed as percentage inhibition, also showed a marked increase, mirroring the TPC trends and highlighting the potential dynamic transformation of bioactive compounds during bacterial growth. For *L. plantarum* IMC 509, OP antioxidant activity rose significantly from 55% to 67% (*p* < 0.001), while OPE increased from 66% to 71% (*p* < 0.001), reflecting the potential release of potent antioxidants. OS and OSE exhibited more moderate non-significant gains. For *L. rhamnosus* IMC 501^®^, OP increased from 56% to 71% (*p* < 0.0001) and OPE from 65% to 70% (*p* < 0.001), while OS and OSE rose from 57% to 61% and 59% to 62%, respectively. The steeper rises with OP and OPE, particularly with the strain IMC 509, underscore the influence of initial phenolic content and strain-specific metabolism, with the most significant enhancements occurring between T0 and T28/30 h.

## 4. Discussion

Phenolic profiles, antioxidant capacity, antimicrobial activity, and probiotic growth-promoting activity of olive oil by-products investigated, namely, OP, OS, and their hydroalcoholic extracts OPE and OSE, revealed their multifunctional potential, underpinned by rich phenolic and mineral profiles. The bacterial cell increments and the growth rate of *L. rhamnosus* IMC 501^®^ and *L. plantarum* IMC 509 demonstrate that all substrates supported probiotic proliferation, with extracts exhibiting better efficacy compared to the raw by-products as such. This observation is consistent with prior research, which attributes enhanced bacterial growth to the increased availability of phenolic compounds following extraction [18]. The liberation of phenolics during hydroalcoholic extraction likely facilitates their microbial utilization [42]. Rather than acting as sole carbon sources, these phenolics are more plausibly co-metabolized alongside the residual medium substrates, and may modulate the culturable population in favour of the inoculated strains [12]. Strain-specific responses, with *L. plantarum* IMC 509 showing greater growth with OPE compared to *L. rhamnosus* IMC 501^®^, suggest metabolic preferences for specific phenolics, as mentioned in a previous study highlighting the interplay between probiotic physiology and dietary bioactives [15]. Moreover, distinctive fermentative behaviors with *L. plantarum* strains have been demonstrated to exhibit distinct metabolic pathways that allow them to effectively degrade certain compounds or produce metabolites under specific conditions. More specifically, in research involving the fermentation of pomegranate juice, *L. plantarum* showed higher sugar consumption and microbial growth compared to other lactic acid bacteria, indicating an efficient fermentation process [43]. This strain-specific adaptability is often linked to the genetic and enzymatic makeup of *L. plantarum*, enabling the utilization of complex substrates and the production of desired outcomes, such as valerate, under optimal conditions [44].

The phenolic profiles provide a chemical rationale for the contrasting bioactivity of the two extracts. In OPE, verbascoside dominated the phenolic fraction, accompanied by secoiridoid derivatives and hydroxytyrosol/tyrosol conjugates, whereas OSE had comparatively less phenolic content and was enriched more in lignans and flavonoids. This distribution agrees closely with the literature on the same matrices; verbascoside and its phenylethanoid derivatives are repeatedly reported as the principal polar phenolics of olive pomace [38,40], while the stone is consistently described as a low-phenolic fraction in which lignans and flavonoids account for a disproportionately large share of a small total pool [39,45]. The higher TPC of OPE (1548.3 ± 443.4 vs. 940.8 ± 127.4 mg GAE mL^−1^) and its correspondingly greater DPPH activity (4365.2 vs. 2788.7 ppm Trolox) mirror values reported for hydroethanolic pomace extracts of comparable cultivars and are in the range expected when verbascoside and secoiridoids are the leading contributors [36].

Besides the achieved probiotic growth promotion, the production of SCFA, particularly acetate (C_2_) and valerate (C_5_), in bacterial culture fluids supplemented with OP, OPE, OS, and OSE highlights their potential to support probiotic growth with fermentative activity. Acetate remained at or above control levels across all four treatments for both strains and was significantly elevated with OP and OPE for *L. plantarum* IMC 509 at T28/30. Acetate is well established as a mediator of colonic barrier integrity and mucosal homeostasis [46], so the maintenance or enhancement of acetate output is consistent with the treatments being compatible with, rather than inhibitory to, probiotic metabolism. Valerate, although less abundant than acetate, followed a clear substrate-dependent pattern: the fiber-rich whole by-products OP and OS elicited the highest C5 output in both strains, whereas the extracts OSE and OPE yielded lower, control-comparable levels. Valerate is well known to contribute to gut health by modulating immune responses, mitigating allergic conditions, and reinforcing intestinal barrier integrity [47,48,49]. Furthermore, C_5_ undergoes β-oxidation in the gut, leading to the production of more prevalent SCFAs, C_2_ and C_3_, as described by Liu et al. (2024) [49]. These smaller SCFAs (C_2_ and C_3_) play a more pronounced role in gut homeostasis, enhancing colonic barrier function, reducing inflammation, and supporting microbial diversity [46]. Taken together, the SCFA data suggest that OP, OPE, OS and OSE are compatible with the fermentative activity of both strains, with the whole by-products favouring valerate and all treatments preserving acetate output; these observations describe probiotic growth-promoting activity compatibility and do not, in themselves, yet establish a prebiotic effect.

The increase in reducing capacity, most marked with OP and OPE, is consistent with probiotic-mediated release or biotransformation of matrix-bound phenolics into more readily oxidizable forms. The significant elevation at T28/30 h, especially with *L. plantarum* IMC 509, is attributable to the potential enzymatic hydrolysis of hydroxytyrosol glucosides, secoiridoids, and verbascoside, leading to an increase in the antioxidant level. This characteristic is crucial in combating oxidative stress in biological systems [50].

The minimal inhibitory concentration (MIC) further elucidates the antimicrobial potential of OPE and OSE against pathogenic strains, with negligible inhibition of probiotic strains. OSE exhibited inhibitory activity, achieving inhibition at low concentrations, particularly against *Ps. aeruginosa* DSM 1117, whilst OPE against *E. coli* ATCC 13706 and *B. cereus* ATCC 9634, a finding attributable to their high phenolic content. The efficacy of phenolics against pathogenic bacteria suggests a potentially specific antimicrobial mechanism, possibly linked to oleacein and verbascoside with their possible degradation products, which are known to disrupt bacterial cell membranes [14].

Mechanistically, olive phenolics are proposed to combine antioxidant protection with direct membrane perturbation, promoting leakage of cellular constituents and heightened susceptibility to environmental stress [51]. While flavonoids can inactivate key microbial proteins involved in adhesion, enzymatic activity, and cell envelope transport, they also potentially disrupt bacterial membranes through the lipophilic nature of flavonoids, leading to perforation, reduced fluidity, and loss of structural integrity [52,53]. In addition, hydroxytyrosol chelates transition metals (iron and copper), forming inert complexes that limit metal bioavailability essential for bacterial growth, while also reducing intracellular ATP levels and causing cell membrane depolarization [54]. Meanwhile, the main potential mechanism by which verbascoside and potential by-products fight pathogenic bacteria is through the inhibition of protein synthesis, specifically by obstructing the uptake of leucine, a key amino acid in protein production, into the bacterial cells, leading to a potential bacteriostatic or lethal effect depending on the concentration and exposure time [55]. The lack of inhibition against *L. plantarum* IMC 509 and *L. rhamnosus* IMC 501^®^ underscores the selective nature of these phenolics, a property consistent with previous studies on phenolic compounds from plant by-products [13]. These results strengthen the case for OPE and OSE as potential natural antimicrobial agents, offering a sustainable alternative to synthetic preservatives in areas of functional foods and pharmaceutical applications [56].

The greater mineral richness of OP relative to OS may contribute to its superior growth compatibility. Potassium, the dominant element in both matrices but proportionally higher in OP, supports probiotic metabolism through osmotic balance and enzyme activation [57]. However, its role in osmotic adaptation varies across bacterial strains, with Le Marrec (2011) noting that while potassium is essential for growth under diverse salinity conditions, it is not always the primary molecule for osmotic regulation in certain *L*. *plantarum* strains [58]. Calcium and phosphorus, present in higher concentrations in OP than OS, are critical for microbial metabolism and structural integrity [59]. The calcium content in OP is comparable to levels reported in previous research about Verdeal and Cobrançosa cultivars, where calcium was a predominant mineral, potentially influencing gut microbiota activity [60]. In the current study, phosphorus, essential for energy transfer and nucleic acid synthesis [61], showed a marked disparity between OP and OS. The latter contained phosphorus at a level that was 22 times lower, whereas magnesium, another key cofactor in enzymatic reactions [62], was also more abundant (about 6 times) in OP than OS, supporting its role in microbial growth [63]. However, the bioavailability and proportional balance of these minerals are critical, as some research noted that their efficacy depends on absorption efficiency, which varies with probiotic strain and dietary context [64]. Strain-specific effects on mineral uptake, as reported by Scholz-Ahrens et al. (2007), further complicate their utilization, necessitating targeted research to optimize probiotic-mineral interactions [65]. Overall, the greater phenolic diversity and concentration of OPE, together with the mineral profile of OP, provide a coherent basis for the bioactivity of the pomace-derived materials.

It should be emphasized that the present design assesses probiotic growth-promoting activity and does not yet constitute a full prebiotic characterization. Establishing a prebiotic effect would require an in vitro model of the intestinal ecosystem demonstrating selective stimulation of host microorganisms that confers a documented health benefit, rather than growth support of pure probiotic cultures in a rich laboratory medium. Future work should also assess how simulated upper-GI digestion alters the phenolic substrates and the viability of the probiotic partners, since digestion can markedly change the effective dose and structure of the bioactives reaching the colon.

Collectively, these findings advocate the valorization of olive by-products as sustainable, multifunctional resources that mitigate environmental burden while offering antioxidant, antimicrobial and probiotic growth-promoting properties. Definitive prebiotic or synbiotic claims, however, await gut-microbiota-based and in vivo validation, alongside assessment of industrial scalability.

## 5. Conclusions

This study demonstrates that OP and OPE exhibit superior probiotic growth-promoting activity and antioxidant properties compared to OS and OSE. The pomace extract was markedly more concentrated in polar phenolics, being dominated by verbascoside (1798.4 ± 123.3 mg L^−1^) and oleacein (948.6 ± 72.5 mg L^−1^), together with hydroxytyrosol and its glucoside (51.9 ± 12.1 and 72.0 ± 1.3 mg L^−1^, respectively) and oleuropein (67.4 ± 21.0 mg L^−1^), whereas the stone extract contained substantially lower levels of these compounds while being comparatively enriched in lignans and flavonoids. Consistently, OPE displayed the higher total phenolic content (1548.3 ± 443.4 vs. 940.8 ± 127.4 mg GAE mL^−1^) and antioxidant capacity (4365.2 vs. 2788.7 ppm Trolox), and the by-products were mineral-rich, most notably in potassium and calcium. Moreover, OPE showed antimicrobial activity against four pathogenic bacteria, with 95% inhibition on *S. aureus* and 69% inhibition on *B. cereus*, while OSE showed up to 97% inhibition on *B. cereus*, and no inhibition was detected on *S. aureus*. These results provide preliminary in vitro evidence supporting further evaluation of olive by-products as natural, antioxidant, antimicrobial ingredients for food applications (e.g., as functional-food additives, natural preservatives or fiber-phenolic-enrichment agents), yet validation in such specific food matrices is the necessary next step. Definitive conclusions regarding prebiotic or synbiotic potential require validation through gut microbiota-based studies and in vivo investigations. Future work should investigate the phenolic biotransformation occurring during fermentation, extend the assessment to gut-microbiota-based models and in vivo systems, and evaluate the technological performance and scalability of these by-product ingredients in real food matrices, thereby supporting their valorisation within a circular-bioeconomy framework.

## Figures and Tables

**Figure 1:**
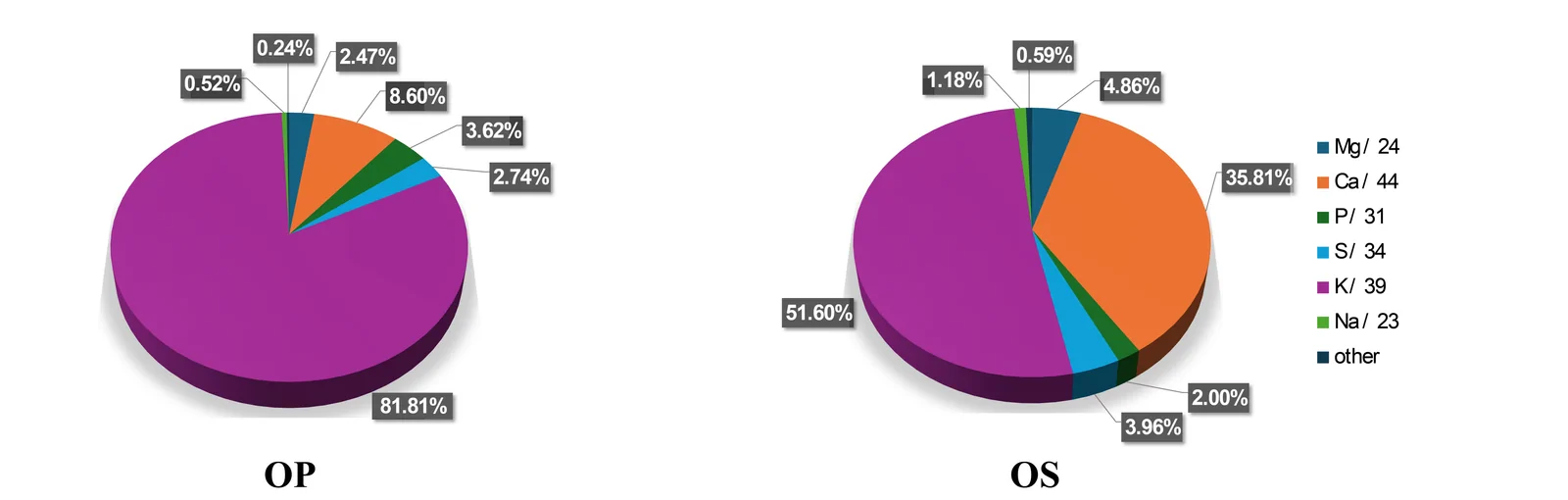
Distribution of the main elements analyzed by ICP-MS present in the Olive Pomace (OP) and Olive Stone (OS) samples.

**Figure 2:**
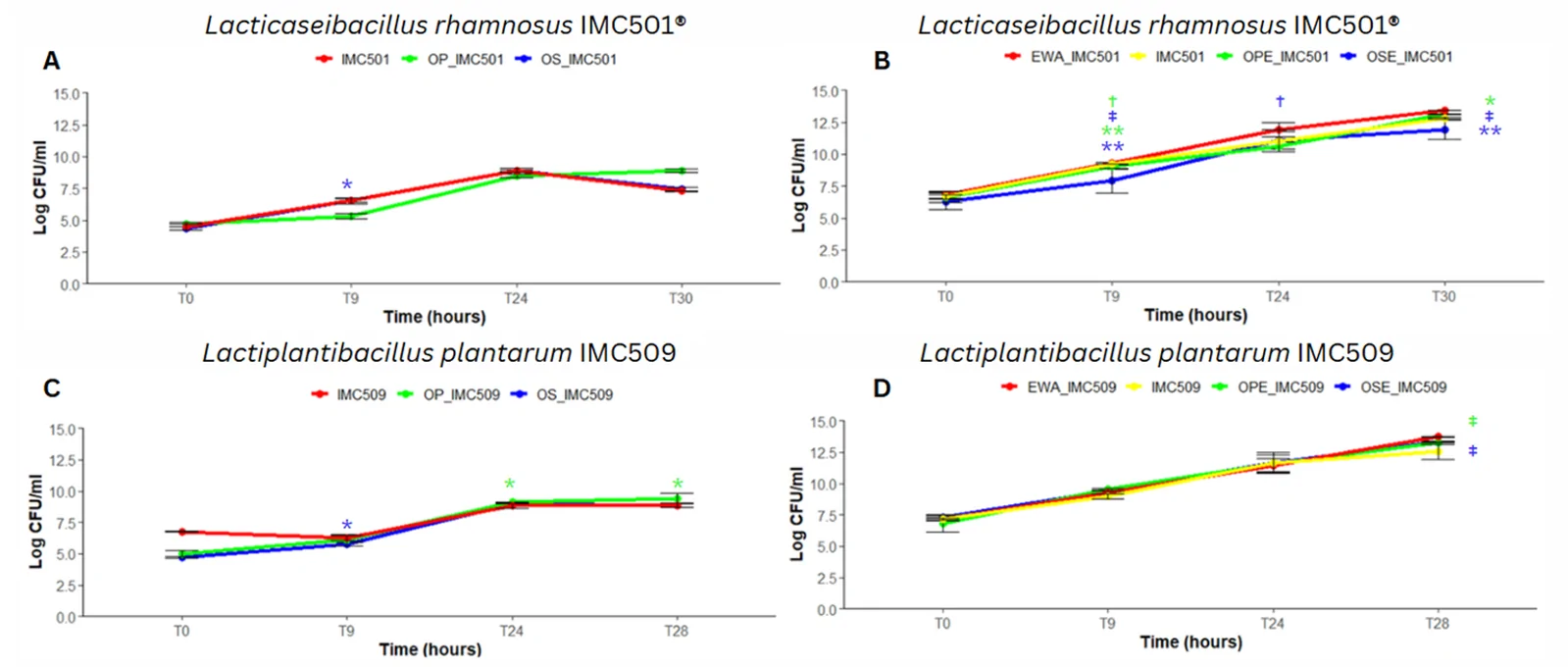
Probiotic bacterial growth curves in the presence of selected substrates, over four time intervals. OP: olive pomace, OS: olive stone, OPE: olive pomace extract, OSE: olive stone extract, EWA: ethanol: water solvent (20:80, *v*/*v*), *L. rhamnosus* IMC 501^®^ and *L. plantarum* IMC 509 control growth. (**A**) Growth of *L. rhamnosus* IMC 501^®^ in the presence of OP and OS. (**B**) Growth of *L. rhamnosus* IMC 501^®^ in the presence of OPE and OSE. (**C**) Growth of *L. plantarum* IMC 509 in the presence of OP and OS. (**D**) Growth of *L. plantarum* IMC 509 in the presence of OPE and OSE. In the graphs, error bars represent standard deviation (*n* = 3), * *p* < 0.05; ** *p* < 0.01, with color representing the substance (OP, OS, OSE, OPE) relation with IMC control; † *p* < 0.05 and ‡ *p* < 0.01, with color representing the treatment (OSE, OPE) in relation to EWA control.

**Figure 3:**
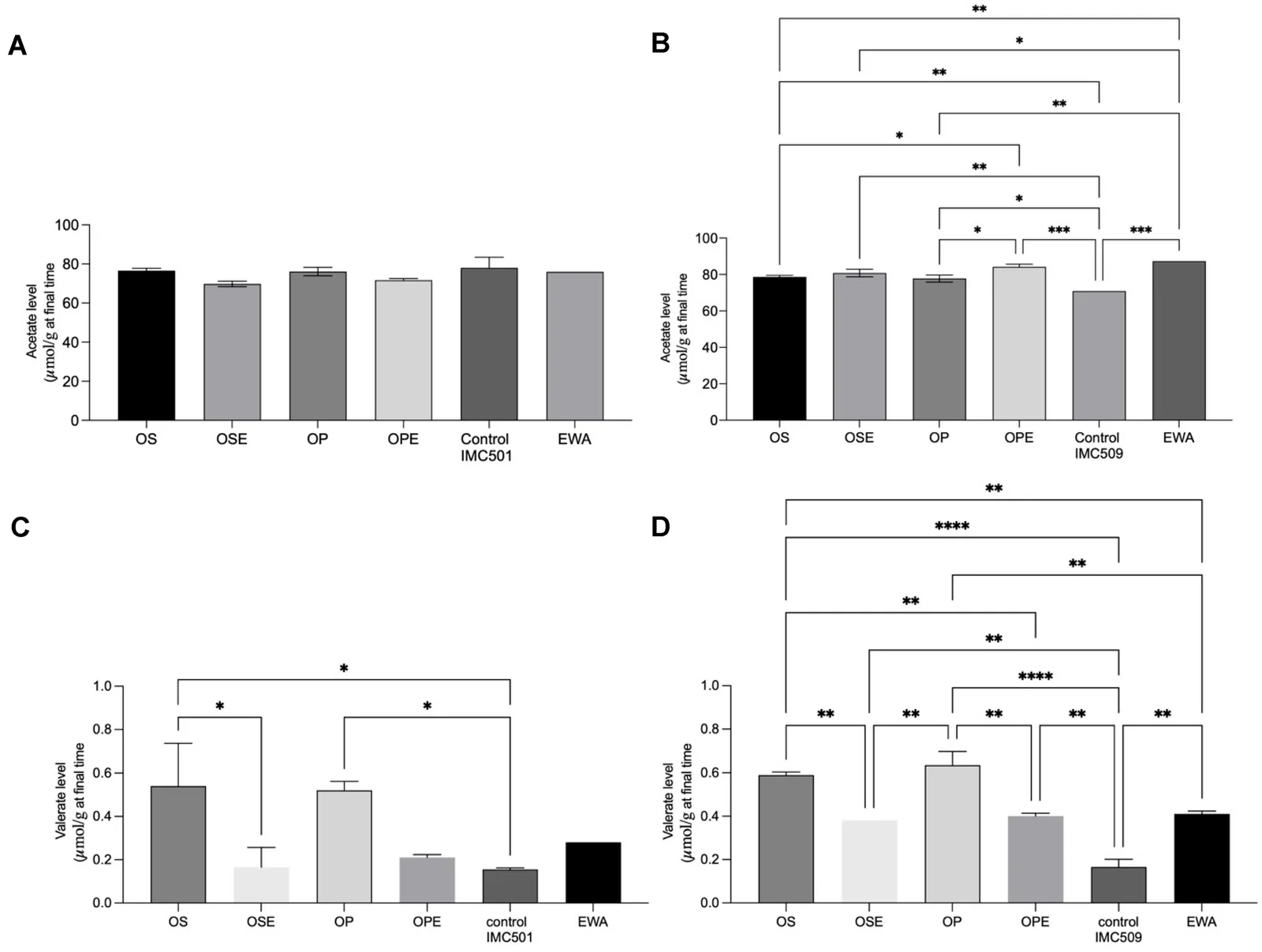
Upper panel: (**A**,**B**): mean acetate (C2) production levels for probiotic strains *L. rhamnosus* IMC 501^®^ and *L. plantarum* IMC 509 with different supplements at the final time of bacterial culture, represented as μmol/g. Lower panel: (**C**,**D**): mean valerate (C5) production levels for probiotic strains *L. rhamnosus* IMC 501^®^ and *L. plantarum* IMC 509 with different supplements at the final time of bacterial culture, represented as μmol/g. Error bars represent standard deviation (*n* = 2). OP: olive pomace, OS: olive stone, OPE: olive pomace extract, OSE: olive stone extract, EWA: ethanol: water solvent (20:80, *v*/*v*), Controls: IMC501 and IMC509 control growth. * *p* < 0.05, ** *p* < 0.001, *** *p* < 0.0001, and **** *p* < 0.00001.

**Figure 4:**
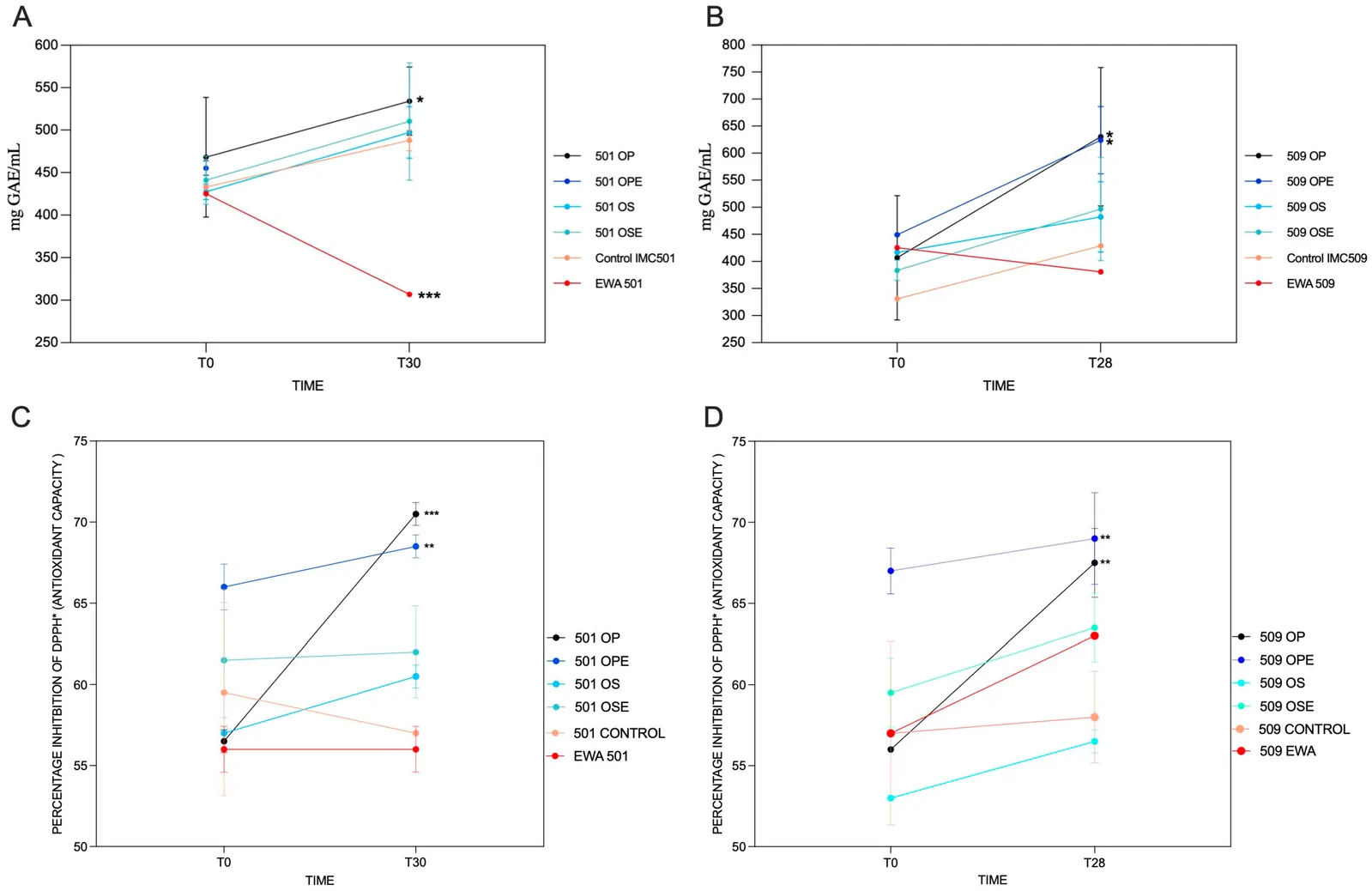
Mean total phenolic content represented as mg GAE/mL in the initial time (T0) and in the final time (T28 or T30) evaluated in bacterial culture fluids of strain *L. rhamnosus* IMC 501^®^ (**A**) and strain *L. plantarum* IMC 509 (**B**) with different supplements and antioxidant levels. Mean percentage inhibition of DPPH, at the initial (T0) and final time (T28 or T30) evaluated in the bacterial culture fluid of strain *L. rhamnosus* IMC 501^®^ (**C**) strain *L. plantarum* IMC 509 (**D**) with different supplements. Error bars represent ± standard deviation (*n* = 2). OP: olive pomace, OS: olive stone, OPE: olive pomace extract, OSE: olive stone extract, EWA: ethanol: water (20:80, *v*/*v*), Control: IMC501 and IMC509 control growth. * *p* < 0.05, ** *p* < 0.001, *** *p* < 0.0001.

**Table 1:** Main phenolic compounds in the olive pomace extract (OPE) and olive stone extract (OSE) solutions analysed by HPLC-DAD/MS, classification according to their chemical family, [M-1]^−^ ion detected by MS analysis, retention time (min), and mean concentration (mg/L) ± standard deviation (SD, *n* = 2), calculated using calibration curves built with the corresponding analytical standard, or standard used as reference, as reported in Section 2.3.1.

Compound Name	Chemical Family	[M-H]^−^	Retention Time (min)	OPE (Mean Concentration mg/L ± SD)	OSE (Mean Concentration mg/L ± SD)
Hydroxytyrosol glucoside	Phenolic alcohol	315	4.09	72.02 ± 1.28	2.43 ± 0.22
Hydroxytyrosol	Phenolic alcohol	153	5.02	51.90 ± 12.10	8.90 ± 0.32
Tyrosol glucoside	Phenolic alcohol	299	5.17	26.00 ± 4.67	ND
Tyrosol	Phenolic alcohol	137	7.73	19.25 ± 3.53	12.18 ± 3.69
Verbascoside	Phenylethanoid glycoside	623	14.95	1798.40 ± 123.34	46.29 ± 5.22
Oleacein	Secoiridoid	319	21.59	948.56 ± 72.51	15.82 ± 8.39
Oleuropein aglycone isomer	Secoiridoid	377	23.22	28.26 ± 18.45	ND
Oleuropein	Secoiridoid	539	24.63	67.44 ± 21.01	ND
Comselogoside	Secoiridoid	535	29.21	11.49 ± 2.46	3.34 ± 0.10
Pinoresinol	Lignan	357	33.35	4.27 ± 0.52	4.37 ± 0.27
Acetoxypinoresinol	Lignan	415	34.22	6.44 ± 0.87	11.29 ± 3.45
Luteolin	Flavonoid	285	35.34	6.83 ± 3.00	11.94 ± 0.79
Oleuropein aglycone isomer	Secoiridoid	377	35.727	10.65 ± 2.15	7.78 ± 4.53
Apigenin	Flavonoid	269	42.331	0.75 ± 0.05	1.09 ± 0.03

ND: not detected.

**Table 2:** Elemental Composition analysis by ICP-MS of olive pomace (OP) and stones (OS). Values are expressed as the average value (mg/kg of sample) ± SD (*n* = 3).

	Olive Pomace (OP) mg/kg ± SD	Olive Stone (OS) mg/kg ± SD
Li/7	0.24 ± 0.09	0.11 ± 0.02
B/11	30.92 ± 11.23	6.20 ± 0.03
Na/23	223.72 ± 7.96	43.59 ± 2.33
Mg/24	1063.75 ± 37.33	179.59 ± 5.69
P/31	1558.54 ± 70.29	73.76 ± 3.63
S/34	1180.40 ± 45.01	146.36 ± 29.27
K/39	35,238.51 ± 560.25	1906.20 ± 57.18
Ca/44	3705.12 ± 102.25	1323.14 ± 32.02
V/51	0.06 ± 0.01	0.03 ± 0.01
Cr/52	0.37 ± 0.01	0.06 ± 0.00
Mn/55	6.02 ± 0.25	1.99 ± 0.09
Fe/56	26.47 ± 1.02	3.21 ± 0.12
Co/59	0.03 ± 0.00	0.002 ± 0.001
Ni/60	0.43 ± 0.02	0.14 ± 0.01
Cu/63	6.83 ± 0.20	1.49 ± 0.04
Zn/66	8.44 ± 0.32	0.85 ± 0.05
Ga/69	0.59 ± 0.04	0.10 ± 0.02
As/75	0.19 ± 0.04	0.04 ± 0.01
Se /78	0.05 ± 0.02	0.017 ± 0.007
Sr/88	9.49 ± 0.32	2.88 ± 0.11
Pd/105	8.11 ± 0.88	3.88 ± 0.29
Cd/111	0.004 ± 0.001	0.0006 ± 0.0001
Cs/133	0.01 ± 0.00	0.004 ± 0.000
Ba/137	4.66 ± 0.25	0.80 ± 0.01
Pb/208	0.06 ± 0.00	0.02 ± 0.00
U/238	0.002 ± 0.000	0.0006 ± 0.0002
Total amount	43,037.01 ± 837.89	3694.47 ± 130.98

**Table 3:** Minimal Inhibition Concentration (mg/mL) and percentage of inhibition of probiotic and pathogenic bacterial strains (*n* = 3). OSE: olive stone extract, OPE: olive pomace extract, EWA: (20:80 (*v*/*v*) ethanol/water), NI—no inhibition.

	OSE		OPE		EWA	
	**Concentration** **(mg/mL)**	**% Inhibition**	**Concentration** **(mg/mL)**	**% Inhibition**	**Concentration** **(ml/mL)**	**% Inhibition**
*L. plantarum IMC 509*	NI		NI		0.1	53.3 ± 0.7
*L. rhamnosus IMC 501^®^*	NI		NI		NI	
*E. coli ATCC 13706*	7.5	55.6 ± 1.5	6.7	76.1 ± 13.4	NI	
*Ps. aeruginosa DSM 1117*	0.2	67.1 ± 7.3	14.0	75.6 ± 23.9	NI	
*S. aureus ATCC 25923*	NI		14.0	95 ± 14.5	NI	
*B. cereus ATCC 9634*	7.5	97.3 ± 28.8	6.7	69.4 ± 0.7	NI	

**Table 4:** Mean values of bacterial cell increment (Δ), expressed as log CFU/mL, the growth rate (µ) and the prebiotic activity score for each probiotic bacterial culture in presence of the several studied substrates.

**Sample**	**Bacterial Cells Increment (Δ)** **(log CFU/mL)**	**Growth Rate (µ)**	**Prebiotic Activity Score**
***L. rhamnosus*** ** IMC 501^®^**			
**OP**	4.216 ± 0.193	0.141 ± 0.006	1.047 ± 0.255
**OS**	3.191 ± 0.192	0.106 ± 0.006	0.079 ± 0.146
**OPE**	7.296 ± 0.132	0.243 ± 0.004	1.179 ± 0.036
**OSE**	6.081 ± 0.016	0.226 ± 0.001	1.105 ± 0.023
**EWA**	6.626 ± 0.126	0.221 ± 0.004	1.076 ± 0.006
***L. plantarum*** ** IMC 509**			
**OP**	4.212 ± 0.050	0.150 ± 0.002	2.067 ± 0.111
**OS**	4.138 ± 0.096	0.148 ± 0.003	2.015 ± 0.183
**OPE**	7.036 ± 0.299	0.251 ± 0.011	1.199 ± 0.066
**OSE**	6.017 ± 0.143	0.215 ± 0.005	1.014 ± 0.003
**EWA**	6.519 ± 0.118	0.233 ± 0.004	1.109 ± 0.236

OP: olive pomace; OS: olive stone; OPE: olive pomace extract; OSE: olive stone extract; EWA: ethanol:water solvent.

## Data Availability

The original contributions presented in this study are included in the article/Supplementary Materials. Further inquiries can be directed to the corresponding author.

## Supplementary Materials

The following supporting information can be downloaded at: https://www.mdpi.com/article/10.3390/foods15193510/s1, Figure S1: Chemical structures and molecular weight (MW, g/mol) of the main phenolic compounds in the olive pomace extract and olive stone extract investigated; Figure S2: Overlaid HPLC-DAD chromatograms recorded at 280 nm of the hydroalcoholic extracts of olive pomace (OPE, red trace) and olive stone (OSE, blue trace). Numbered peaks correspond to phenolic compounds identified by HPLC-DAD-MS on the basis of their deprotonated molecular ions [M–H]^−^: (1) hydroxytyrosol glucoside (*m*/*z* 315); (2) hydroxytyrosol (*m*/*z* 153); (3) tyrosol glucoside (*m*/*z* 299); (4) tyrosol (*m*/*z* 137); (5) verbascoside (*m*/*z* 623); (6) oleacein (*m*/*z* 319 [M–H]^−^; 639 [2M–H]^−^); (7) oleuropein aglycone isomer (*m*/*z* 377); (8) oleuropein (*m*/*z* 539); (9) comselogoside (*m*/*z* 535); (10) pinoresinol (*m*/*z* 357); (11) acetoxypinoresinol (*m*/*z* 415); (12) luteolin (*m*/*z* 285); (13) oleuropein aglycone isomer (*m*/*z* 377). Apigenin was not detected at 280 nm.
