# Supplementary material for: Elucidating the Multifunctional Roles of Olive Oil By-Products: Phenolic Profiles, Antioxidant Capacity, Probiotic Growth-Promoting and Antimicrobial Activity
Source: Foods. 2026 Oct 1;15(19):3510. doi: 10.3390/foods15193510 (PMC13664482; doi:10.3390/foods15193510)
Supplement: Supplementary file 1 [file foods-15-03510-s001.zip › foods-4551178-supplementary.pdf]

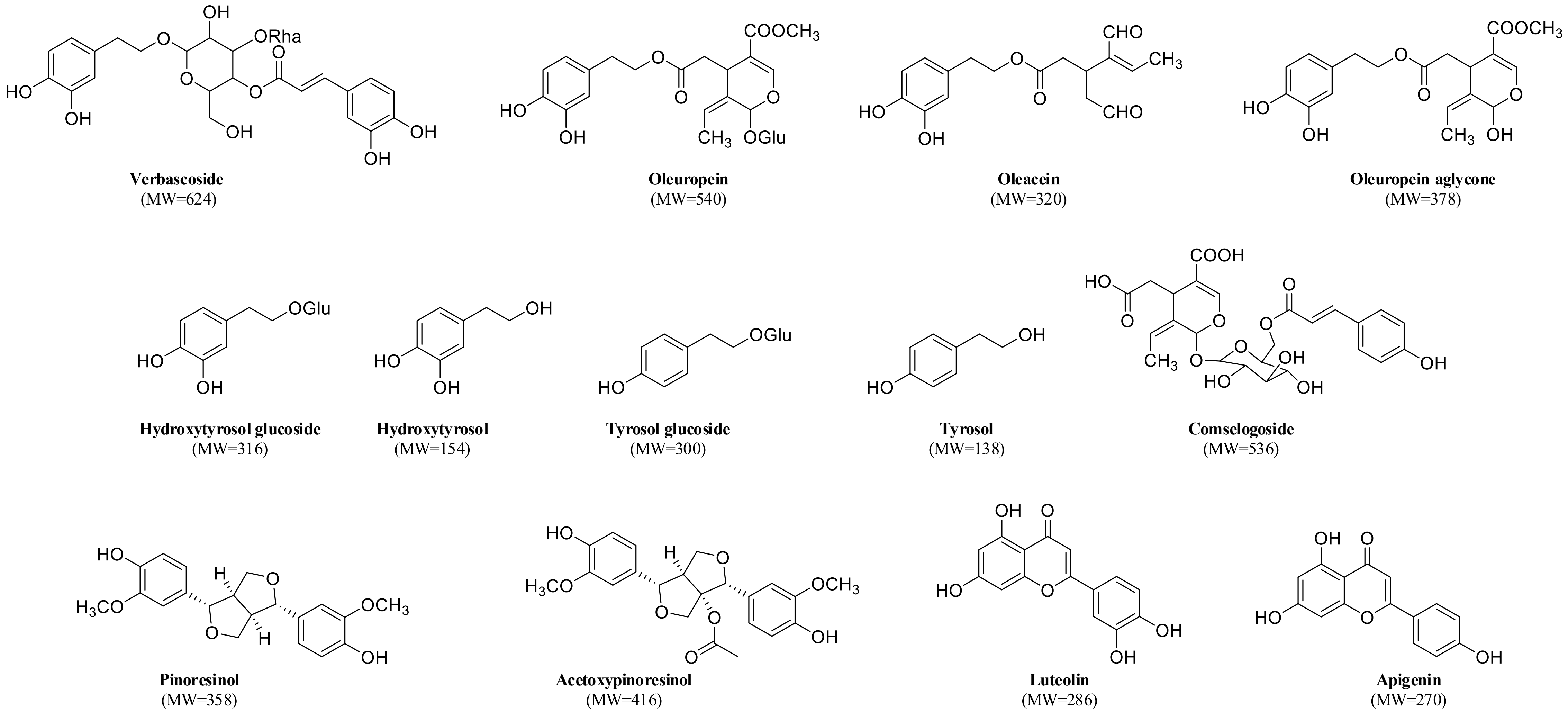

Figure S1. Chemical structures and molecular weight (MW, g/mol) of the main phenolic compounds in the olive pomace extract and olive stone extract investigated.

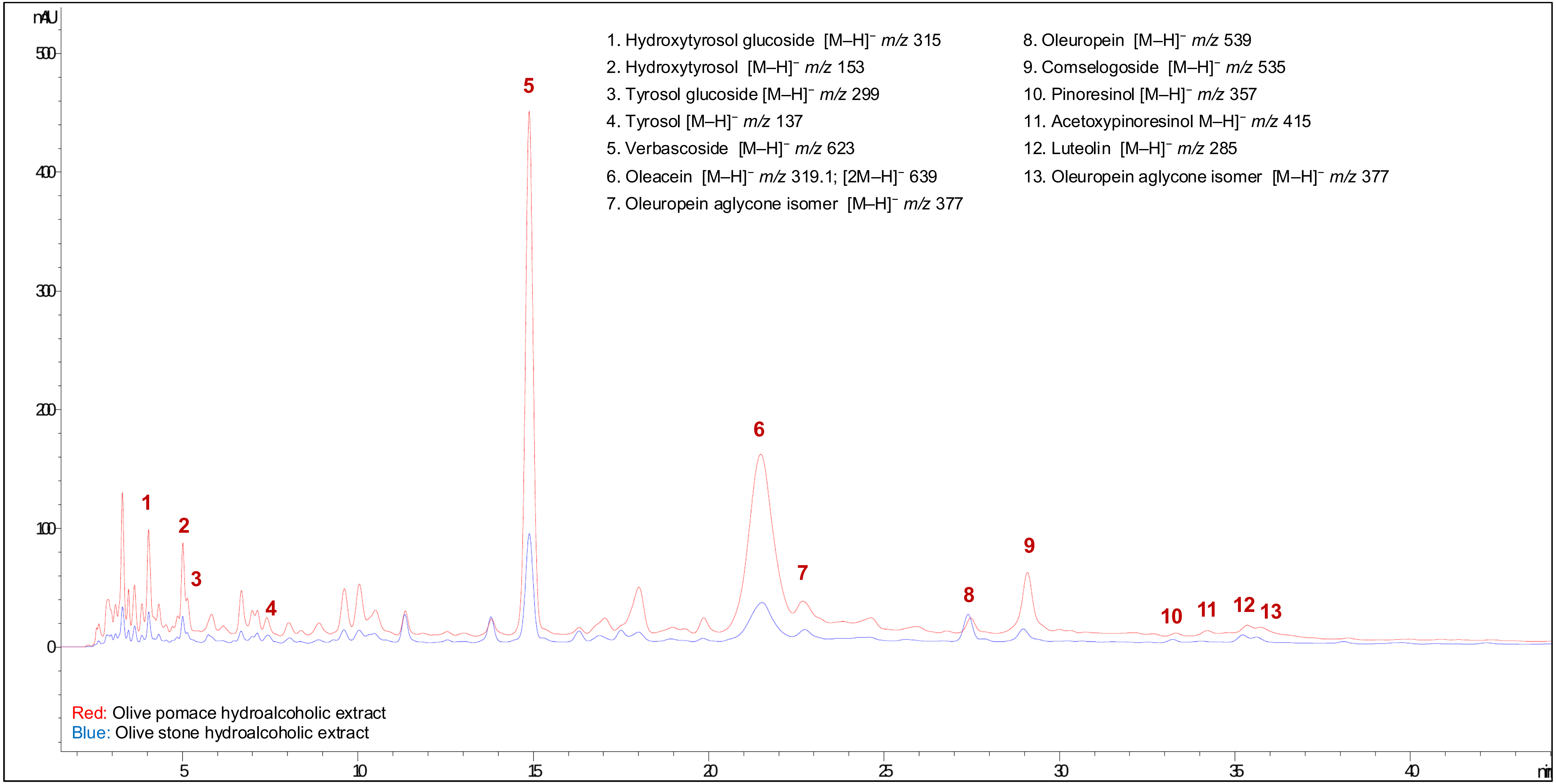

Figure S2. Overlaid HPLC-DAD chromatograms recorded at 280 nm of the hydroalcoholic extracts of olive pomace (OPE, red trace) and olive stone (OSE, blue trace). Numbered peaks correspond to phenolic compounds identified by HPLC-DAD-MS on the basis of their deprotonated molecular ions $[M–H]^-$: (1) hydroxytyrosol glucoside (m/z 315); (2) hydroxytyrosol (m/z 153); (3) tyrosol glucoside (m/z 299); (4) tyrosol (m/z 137); (5) verbascoside (m/z 623); (6) oleacein (m/z 319 $[M-H]^-$; 639 $[2M–H]^-$); (7) oleuropein aglycone isomer (m/z 377); (8) oleuropein (m/z 539); (9) comselogoside (m/z 535); (10) pinoresinol (m/z 357); (11) acetoxypinoresinol (m/z 415); (12) luteolin (m/z 285); (13) oleuropein aglycone isomer (m/z 377). Apigenin was not detected at 280 nm.
